# A Network Approach to Compliance: A Complexity Science Understanding of How Rules Shape Behavior

**DOI:** 10.1007/s10551-022-05128-8

**Published:** 2022-05-10

**Authors:** Malouke Esra Kuiper, Monique Chambon, Anne Leonore de Bruijn, Chris Reinders Folmer, Elke Hindina Olthuis, Megan Brownlee, Emmeke Barbara Kooistra, Adam Fine, Frenk van Harreveld, Gabriela Lunansky, Benjamin van Rooij

**Affiliations:** 1 School of Law, University of Amsterdam, Roetersstraat 11, 1018 WB Amsterdam, The Netherlands; 2 Department of Psychology, University of Amsterdam, Nieuwe Achtergracht 129-B, 1018 WS Amsterdam, The Netherlands; 3 National Institute for Public Health and the Environment (RIVM), Antonie van Leeuwenhoeklaan 9, 3721 MA Bilthoven, The Netherlands; 4 School of Criminology and Criminal Justice, Arizona State University, University Center, 411 N Central Ave, #600, Phoenix, USA; 5 School of Law, University of California, Irvine, 401 E Peltason Dr Suite 1000, Irvine, CA 92697 USA

**Keywords:** Network analysis, Compliance, Complexity science

## Abstract

To understand how compliance develops both in everyday and corporate environments, it is crucial to understand how different mechanisms work together to shape individuals’ (non)compliant behavior. Existing compliance studies typically focus on a subset of theories (i.e., rational choice theories, social theories, legitimacy theories, capacity theories, and opportunity theories) to understand how key variables from one or several of these theories shape individual compliance. The present study provides a first integrated understanding of compliance, rooted in complexity science, in which key elements from these theories are considered simultaneously, and their relations to compliance and each other are explored using network analysis. This approach is developed by analyzing online survey data (*N* = 562) about compliance with COVID-19 mitigation measures. Traditional regression analysis shows that elements from nearly all major compliance theories (except for social theories) are associated with compliance. The network analysis revealed groupings and interconnections of variables that did not track the existing compliance theories and point to a complexity overlooked in existing compliance research. These findings demonstrate a fundamentally different perspective on compliance, which moves away from traditional narrow, non-network approaches. Instead, they showcase a complexity science understanding of compliance, in which compliance is understood as a network of interacting variables derived from different theories that interact with compliance. This points to a new research agenda that is oriented on mapping compliance networks, and testing and modelling how regulatory and management interventions interact with each other and compliance within such networks.

**Supplementary Information:**

The online version contains supplementary material available at 10.1007/s10551-022-05128-8.

## Introduction

Corporate compliance is a key challenge and a key aspect of the practice and study of business ethics. There is a large literature on what variables shape compliance. Part of this literature has sought to understand compliance at the corporate organizational level. Here, much attention has been devoted to corporate compliance management systems, codes of conduct, and whistleblower complaint systems. Studies have looked at whether these compliance management systems are effective (Coglianese & Lazer, 2003; McKendall et al., 2002; Weaver et al., 1999), or what aspects of such systems are effective in ensuring compliance (Parker & Nielsen, 2009). A recent review of this body of work concludes that compliance management systems only result in modest improvements in risk reduction, and that for such systems to be effective, they need to exist in a favorable organizational culture with support from management and information technologies (Coglianese & Nash, 2021).

However, both in corporate and everyday settings, (non)compliance ultimately involves individuals who obey or break rules, laws, or policies. As such, understanding the processes that shape individual (non)compliance is essential in order to effectively address compliance risks. Research in corporate settings is particularly oriented towards workplace unethical behaviors, which different studies have associated with a range of different variables, situated in individual characteristics, moral issue characteristics, and organizational environment characteristics (for a meta-analysis, see Kish-Gephart et al., 2010). Among others, different studies have looked at personality factors (e.g.,Trevino & Youngblood, 1990), incentives (e.g., Ashkanasy et al., 2006), justice considerations (e.g., Trevino & Weaver, 2001), social processes (e.g., O’Fallon & Butterfield, 2012; Thau et al., 2015), and opportunities for offending (e.g., Pendse, 2012). Broadening the perspective towards the broader social and behavioral sciences, there is an even larger and more diverse body of work that has examined why individuals comply with particular rules, laws, or policies. In this literature, we can distinguish five key theoretical approaches, which have developed in relative isolation from each other—in different disciplines, focusing on different settings and behaviors. Broadly they comprise rational choice theories (Becker, 1976; Shover & Hochstetler, 2005), social theories (Nolan & Wallen, 2021; Schultz et al., 2007), legitimacy theories (Murphy & Tyler, 2008; Tyler, 1997, 2006; Tyler & Blader, 2005), capacity theories (Langton & Piquero, 2007; Pratt & Lloyd, 2021; Van Rooij, 2021), and opportunity theories (Benson & Madensen, 2007; Benson et al., 2009; Clarke, 2003). Each of these literatures has focused on its own concepts and variables to understand how these predict individual compliance within specific settings. However, the literature lacks an integrated perspective that illuminates how this spectrum of variables interacts with individual compliance in particular settings in corporate or everyday life. Indeed, Kish-Gephart et al. (2010) signal an urgent need for research that simultaneously considers different explanations.

The present study seeks to move beyond the siloed approach to compliance that has dominated existing studies so far. It is premised on the notion that compliance is a multifaceted phenomenon that is likely related to a multitude of factors from across the existing five theoretical domains. Most likely the influences on compliance do not operate independently from each other, but rather will show complex interrelationships. Consider for instance punishment, one of the key variables in a rational choice approach to compliance. Enhancing punishment may not only affect the perceived cost of noncompliance, but also may crowd out social norms (a key aspect of social theories) that strengthen compliance (Gneezy & Rustichini, 2000), or convey negative social norms by suggesting that offending is common (Cialdini et al., 2006). Punishment may also interact with people’s capacity to comply, in that it may be ineffective for people who do not know the rules (Darley et al., 2001); moreover, punishing offenders may take away their ability to lead law-abiding lives, for example by impeding their access to work or housing (Alexander, 2010). Moreover, compliance itself may affect the factors that come to shape it: for instance, the frequency of offending may also impact expectations of punishment, and actual punishment levels (Bar-Gill & Harel, 2001). As this example illustrates, it is likely that key mechanisms of individual compliance do not operate independently, but rather show complex relationships that are obscured in the narrow approaches that have dominated existing research.

For this reason, the present study seeks to understand individual compliance as part of a complex system in which a multitude of variables interact with each other and with compliance (Barabási, 2007, 2016). From a complexity approach (Cilliers, 2000; Meadows, 2008), compliance would be part of a larger, interconnected and interacting system of relevant mechanisms that cannot be fully understood by isolating specific variables, or a subset thereof. Conversely, if the complex system in which compliance is embedded is not properly understood, compliance interventions may have unforeseen results. The present study draws on the network analysis method developed in complexity science, as applied also in psychology (Barabási, 2007; Borsboom & Cramer, 2013; Borsboom et al., 2021; Dalege et al., 2018; van der Maas et al., 2020). This approach allows us to understand individual compliance in relation to the key theoretical approaches from the social and behavioral sciences, as well as illuminating the relationships between their focal concepts.

The study is highly relevant for corporate compliance and business ethics for several reasons. First, it responds to the call for greater integration of explanations of individual compliance (Kish-Gephart et al., 2010). It does so by situating individual compliance in the broadest and most extensive bodies of knowledge on the subject. Second, with the network approach we employ here, it provides a template for studying these processes across different specific settings in corporate or everyday life. Third, by demonstrating how these variables interrelate in a complex network, the network approach to compliance showcased here can be further developed to help to illuminate how individual compliance may be shaped by particular compliance interventions (e.g., by modeling their direct and indirect effects in the network in simulations, see Lunansky et al., 2021).

The present paper applies this network approach to individual-level compliance through a study of behavioral responses to virus mitigation measures during the first wave of the COVID-19 pandemic. Governments around the world introduced these measures to curb the spread of the virus, affecting both individuals and corporations by placing far-reaching restrictions on individual behavior. This setting is suitable for applying a network perspective on individual compliance because it concerns the introduction of a novel set of rules which applied to all individuals; as opposed to many other rules, laws or policies which apply to subsets of individuals or companies, and where responses have already become habitual. Recent work has shown that empirical network models can provide insight into the interplay of psychological factors that are important in relation to COVID-19-related behavior (Chambon et al., 2021, 2022; Taylor et al., 2020). Furthermore, it represents a setting in which core variables from all five major compliance theories have been hypothesized to be at play (for a review, see Kooistra & van Rooij, 2020; also see Reinders Folmer et al., 2021). These features make this a setting that is ideally suited for understanding how these variables may interrelate with individual compliance in a complex network. With our theoretical focus on individual compliance, as well as our network approach, our research also moves beyond such related work, which has principally focused on pandemic mitigation, and failed to consider the complex relationships between predictors.

For these purposes, our study leverages a survey that operationalizes variables from across the five key compliance theories. To demonstrate the contribution of our network approach, we first rely on traditional statistical analyses (correlational and regression), and then conduct network analysis in which all variables are modeled as an interconnected network. From the results, we explain how the network understanding of individual compliance differs from the insights obtained from traditional non-network statistical approaches. Furthermore, we discuss how the observed network aligns with, or differs from the original, siloed theoretical approaches. Based on this, we advance a template for a network approach to individual compliance, with which these processes can be modeled in other (corporate or everyday) settings. The purpose of the paper therefore is methodological and theoretical: it seeks to showcase a network approach to individual compliance within a specific sample and empirical setting. We do not claim that the observed compliance network within the empirical setting studied here will directly generalize to other samples, or to other settings in everyday or corporate life. Rather, the study employs this empirical setting to demonstrate how a network approach can advance our understanding of individual compliance here, and to draw out implications and recommendations for studying compliance in other (corporate or everyday) settings.

### Compliance Theories

The study of compliance focuses on the interaction between legal rules, and human and organizational conduct. While some studies take an interpretative and endogenous approach, in seeking to understand how behavioral responses to the law shape the meaning of legal rules (Edelman, 1992; Edelman & Talesh, 2011; Edelman et al., 1991; Lange, 2002), most studies of compliance seek to understand why people obey or break rules. This latter question has been studied across different academic domains, across different types of rules, and with a focus on different mechanisms and interventions that may shape behavioral responses to legal rules (van Rooij & Sokol, 2021). This has resulted in a patchwork of different theories that are seldomly brought together and that exist in compartmentalized silos, each with their own literatures, methods and findings. The present study seeks to make use of some of the most important ideas that have developed across these different literatures to study compliance with COVID-19 mitigation measures in the context of the Netherlands as it occurred during the first pandemic wave.

We can distinguish several families in compliance theories. The first family are the *rational-choice theories*. These have developed in economics (Becker, 1968) and criminology (Gul, 2009; Shover & Hochstetler, 2005). According to this theoretical approach, compliance originates in a rational choice, where, simply summarized, actors will choose to break the rules if the benefits (minus costs) of compliance are lower than the benefits (minus costs) of violation. As such, a first aspect of rational choice theories is the cost of compliance (Donovan & Blake, 1992; Paternoster & Simpson, 1993). Such theories hold that fewer people will comply when the costs of compliance are higher than the costs of noncompliance. In the context of COVID-19 mitigation, we thus expect that when the costs of complying with mitigation measures are higher (for instance when people lose income or their job), compliance will decrease. Related to this are the benefits of compliance (although there is less study of this). In context of COVID-19, these particularly relate to the mitigation of the threat of the virus. Based on rational choice theories, we thus expect that the more people fear the threat of the disease (and thus value mitigating it), the more they will comply.

A second key aspect of these theories (and the aspect that has attracted most scholarship) is general deterrence: the costs that people perceive they may suffer should they break the rules. According to general deterrence theory, people comply more with rules when there is a greater certainty and severity of punishment (Becker, 1968; Polinsky & Shavell, 2000; Shavell, 1991). There is no conclusive evidence that stronger punishment alone deters people from crime (Braga et al., 2019; Schell-Busey et al., 2016; Simpson et al., 2014); there is stronger evidence that more certain punishment does so (Nagin, 2013). However, research shows that deterrence is a subjective mechanism (Apel, 2013; Decker et al., 1993), and, therefore, that it is important to study the perceptions that people have of both the certainty of punishment and the impact that punishment may have on their lives (Grasmick & Bryjak, 1980). Thus, following deterrence theory, we expect that greater perceived certainty of punishment and greater perceived severity of punishment will have a deterrent effect, and therefore will result in greater compliance with mitigation measures.

A second family of compliance theories is oriented on the social embeddedness of human conduct and human responses to the law. These *social theories* have developed for instance in social norm theories in psychology (Nolan & Wallen, 2021; Nolan et al., 2008; Schultz et al., 2007), and social learning theories in criminology (Akers & Jensen, 2011; Pratt et al., 2010). These theories hold that human behavior is not merely driven by motivational forces on the individual level (as rational choice at least implicitly seems to hold), but that behavior and its meaning is deeply embedded in a social context. As a result, the more that one’s social context is opposed to legal rules, the more likely it is that there will be non-compliance. Psychological research has found that the more that people see others violate rules (i.e., a descriptive social norm for not complying), the more likely they are to do so themselves (Cialdini & Trost, 1998; Cialdini et al., 2006; Goldstein et al., 2008; Schultz et al., 2007). Thus, based on social theories, we expect that the more that people see others not following COVID-19 mitigation measures, the less that they will do so themselves. Conversely, the more that others are seen to comply, the more that they themselves will do so too.

A third family of theories look at the *legitimacy* of the rules, the authorities, or the law in general. Building on Tyler’s foundational work (Tyler, 2006), a large body of research has shown that people’s responses to legal rules are associated with their perceptions of those rules, and of the authorities that adopt and enforce them. Generally, these studies find that the more people see the rules and the authorities as legitimate, the more they will comply with them (Murphy & Tyler, 2008; Nagin & Telep, 2017; Tyler, 1997, 2006, 2017; Tyler & Blader, 2005; Walters & Bolger, 2019). Here, we can distinguish between a substantive legitimacy, where people’s own morals and preferences are in line with the substance of the rules (or simply put: where they agree with the rules), and a procedural legitimacy, where people perceive the procedures through which the rules were adopted and implemented as fair and just (Tyler, 1997, 2006). Both forms of legitimacy are associated with compliance: the more people agree with rules, the more they comply (Tyler, 1997, 2006), and the more they see rules and their implementation as procedurally fair, the greater their compliance (Walters & Bolger, 2019). Closely related to legitimacy is the obligation to obey the law (“OOL”). This refers to people’s felt obligation to obey the law in general, simply because it is the law, even when there is limited enforcement, or when they do not feel a particular social or moral reason to follow the rules (Fine & Van Rooij, 2021). OOL is a core expression or a downstream consequence of people’s felt legitimacy, as people with a higher sense of legitimacy will develop a higher OOL when they view authorities as legitimate (Gau, 2015). People who have a higher OOL are more likely to comply with the law (Tyler, 2006). As such, based on legitimacy approaches to compliance, we expect that people will comply more with COVID-19 mitigation measures the more they morally support these measures; the more they support the authorities and their policy response to the virus; the more they perceive the enforcement of these rules to be procedurally fair; and the more they feel a general duty to obey the law.

A fourth family of compliance theories concerns the *capacity* people have to comply with the rules. It stands to reason that the more difficult it practically is for people to comply with rules, the less likely they will be to effectively do so. For people to have the capacity to comply, it is important they have sufficient knowledge of what is expected of them (Darley et al., 2001; Kim, 1999; Van Rooij, 2021). However, research demonstrates that people’s knowledge of legal rules is often inaccurate or lacking, reducing their compliance (Van Rooij, 2021). Therefore, we expect that people with more knowledge of mitigation measures will show greater compliance. Additionally, the more unclear rules are to people, the more difficult it is for them to know what is expected of them (Feldman & Teichman, 2009). For this reason, we expect that people who experience the mitigation measures as less clear will display lower compliance. A related aspect of people’s capacity to follow rules is whether they are able to exert self-control, and are able to restrain themselves from breaking rules. Previous criminological findings have shown that high levels of impulsivity predict deviant behavior (Gottfredson & Hirschi, 1990; Pratt & Cullen, 2000, 2005; Pratt & Lloyd, 2021; Vazsonyi et al., 2017). Related to this, some people’s capacity to follow the rules may be undermined by negative emotions they experience or develop. More specifically, a considerable body of research on strain theory has demonstrated an association between negative emotions and rule violating and deviant behavior, such that people may cope with negative emotions through rule-breaking (Agnew, 1992, 2007; Agnew & White, 1992; Agnew et al., 2002; Baron, 2004; Botchkovar et al., 2009; Langton & Piquero, 2007; Piquero & Sealock, 2004). Accordingly, we expect that compliance with mitigation measures will be lower among more impulsive people, and among people who experience more negative emotions as a result of the pandemic. More generally, capacity theories thus imply that people will comply more with mitigation measures the more they are able to do so (Reinders Folmer et al., 2021).

The fifth, and final theoretical family that we incorporate focuses on opportunities people have for breaking the rules. A large body of work from criminology (Benson & Madensen, 2007; Benson et al., 2009; Van Rooij & Fine, 2021) and behavioral ethics (Feldman, 2018) has shown that compliance is shaped by the situation in which it takes place. Routine activities theory has for instance shown that criminal behavior develops more easily when there are attractive targets which are left undefended to motivated offenders (Cohen & Felson, 1979; Osgood et al., 1996; Spano & Freilich, 2009). Situational crime prevention theory has broadened this idea toward all situations that lower the threshold for illegal behavior, for instance by providing easy access to tools or techniques needed to break the law (Clarke, 2003, 2005). Based on these theories, we thus expect that the more opportunities people have to violate COVID-19 mitigation measures, the less they will comply.

### The Present Study

The present study seeks to understand individual compliance as part of a complex system in which a multitude of variables interact with each other and with compliance to shape one another (Barabási, 2007, 2016). Our approach aims to situate individual compliance in relation to the five major theoretical approaches to the subject, as well as to illuminate the relationships between their focal concepts. We develop our approach in the setting of COVID-19 mitigation measures, during the first pandemic wave in the Netherlands. For this purpose, we conducted a survey that assessed compliance with social distancing and stay-at-home measures, as well as key mechanisms from each of the major compliance theories (see Table 1).

**Table 1:** Overview of compliance theories and mechanisms

Compliance theories	Mechanism	Submechanism
*Rational choice theories*		
	Costs of compliance	
	Perceived threat	
	Deterrence	
		Certainty of punishment
		Severity of punishment
*Social theories*		
	Social norms	
*Legitimacy theories*		
	Moral support for measures	
	Support for policies	
	Procedural justice	
	Obligation to obey the law (OOL)	
*Capacity theories*		
	Capacity to comply	
	Knowledge of measures	
	Clarity of measures	
	Impulsivity	
	Negative emotions	
*Opportunity theories*		
	Opportunity to violate	

Our approach draws on network analysis, which empirically estimates network models through statistical analysis (see Dalege et al., 2017 for a tutorial in the context of attitudes). A network is a graphical representation of the measured variables (*nodes*) and the links or relationships (*edges*) between them (Dalege et al., 2017; Hevey, 2018). As such, network analysis allows us to understand the system-level relationships of the compliance theories and their potential mechanisms (Hevey, 2018).

In the estimation of the network model, edges only appear after controlling for every other node in the network (Epskamp et al., 2018c). This means that the specific relation between two nodes cannot be explained by the presence of other variables. A positive edge between two nodes indicates their preference to align (Epskamp et al., 2018a). For example, suppose that the variables punishment certainty and punishment severity would be positively connected. This would indicate that people who, on average, perceive punishment to be more likely, also, in general, perceive punishment to be more severe. Contrary, a negative edge indicates a negative association between connected nodes. A graphical illustration of an estimated network model can illustrate how concepts from the different theoretical approaches may relate to compliance and to each other—and thereby, may provide insight into the potential mechanisms related to compliance.

Furthermore, network analysis provides insight into *clusters*, or nodes that are highly interconnected among themselves, but poorly connected with nodes outside this cluster (Hevey, 2018). As such, a network approach will demonstrate whether factors from particular theoretical families will form distinct clusters, as would be expected based on their distinct literatures. In addition, network analysis will provide insight into *centrality*, or the extent to which nodes are more (or less) connected in the network, and hence more (or less) important (Borgatti, 2005; Freeman, 1978). The hypothesis is that more centrally located nodes may have a larger influence on the network’s behavior (Epskamp et al., 2018a). A clear hierarchical structure in node centrality may potentially favor a particular compliance theory, for instance when several variables of a theory show high centrality. Last, to demonstrate how a network approach moves beyond traditional, non-network approaches to compliance, we contrast these insights with those obtained through correlational and regression analysis.

Our contribution to the existing literature is threefold. First, our network approach enables us to understand the interrelations that exist between the different theoretical concepts that are obscured in a traditional, non-network approaches to compliance. This enables us to understand how changes in particular variables may spread throughout the network to directly or indirectly impact other variables, including compliance. Secondly, our network approach allows us to assess if the observed structure of variables aligns with the major compliance theories, such that variables originating from the same theory form distinct clusters, which are separate from variables from other theories. This comparison allows us to understand whether it makes empirical sense to approach compliance from these singular theories, or whether it is more appropriate to integrate them, in ways that may not previously have been considered in the literature. Finally, our research contributes to the general literature on compliance by providing a template for a complex system approach to compliance, with which compliance can be studied in other settings in corporate or everyday life.

## Methods

Ethical approval was obtained from the Ethics Review Board of the Amsterdam Law School of the University of Amsterdam on April 3, 2020. All participants provided consent before taking part in the study. Participation was voluntary, and participants could stop the survey at any time.

### Participants

Participants were recruited between April 7 and April 14, 2020, through the online platform Prolific Academic. They were redirected to Qualtrics to fill out a survey (in English). Only English-speaking residents of the Netherlands^1^ aged 18 years or higher were allowed to participate. They were paid 2.44 GBP for participating. The initial sample consisted of 614 participants, of whom 32 were excluded for not finishing the survey. Furthermore, eight participants were excluded because they provided professional care for COVID-19 patients, and seven participants because they failed the attention check.^2^ Finally, six participants indicated a non-binary gender orientation—an insufficient number to study as a separate category; hence, they were also omitted. This resulted in a final sample of *N* = 562. Table 2 shows the sample characteristics.

**Table 2:** Sample characteristics

Characteristic	Mean (SD)	Percentage	Scale
Age	27.57 (8.52)		18–100
Gender			
Female		44.7%	
Male		55.3%	
Employed			
Yes		51.6%	
No		48.4%	
Education			
No diploma		1.2%	
High school degree		35.8%	
College degree and higher		63.0%	
Ethnic minority			
Yes		16.7%	
No		81.3%	
Socio-economic status	6.34 (1.56)		1–10
Household size	2.79 (1.54)		0–25
Health risk self			
Yes		13.0%	
No		87.0%	
Health risk others			
Yes		74.6%	
No		25.4%	
Trust in science	4.29 (0.80)		1–5
Trust in media	2.93 (1.11)		1–5

Employed—yes = full-time, part-time, or self-employed; no = unemployed, student, retired, homemaker, unable to work

### Materials

Table 3 shows the number of items and a sample item for all dependent and independent variables. A detailed description of these materials can be found in “Appendix A”.

**Table 3:** Number of items and examples for dependent and independent variables

Variables (no. items)	Example of item
Compliance (5)	I still meet people outside of my direct household
Rational choice theories	
Costs of compliance (5)	Due to the measures to contain the Coronavirus, I will likely lose income
Perceived threat (3)	I believe the Coronavirus is a major threat to my health
Punishment certainty (4)	How probable is it that authorities will punish you if you do not follow social distancing measures?
Punishment severity (2)	How much will you suffer if authorities punish you not following social distancing measures?
Social theories	
Social norms (5)	Most people I know are following social distancing measures
Legitimacy theories	
Moral support (2)	I morally believe that people should follow social distancing measures to contain the Coronavirus
Support for policies (2)	Authorities and government officials have been consistent with their approach to contain the Coronavirus
Obligation to obey the law (1)	I feel like it is sometimes okay to break the law
Procedural justice (7)	In enforcing the measures to reduce the spread of the Coronavirus, I expect that the authorities will treat people with respect
Capacity theories	
Capacity to comply (3)	At this moment, I am able to keep a safe distance from others
Knowledge of measures (7)	According to measures adopted by authorities to contain the Coronavirus, I am currently required to not meet people from my direct household
Clarity of measures (1)	The measures authorities have adopted to reduce the spread of the Coronavirus are: (extremely unclear–extremely clear)
Impulsivity (5)	I should try harder to control myself when I’m having fun
Negative emotions (6)	The Coronavirus makes me feel angry
Opportunity theories	
Opportunity to violate (5)	At this moment, if it were against the rules, it would still be possible for me to meet people outside of my direct household

### Analyses

We first examined the associations between compliance and the theoretical variables using traditional, non-network analyses (i.e., correlations and regression analysis). Then, we conducted network analysis to understand their system-level relationships.

#### Correlations and Regression Analyses

Preliminary data analyses revealed negative skew in the dependent variable and heteroskedasticity. For these reasons, we relied on nonparametric correlations, and on regression analyses with robust (heteroscedasticity-consistent) standard errors. Nonparametric correlations (Kendall’s tau) between compliance and additional variables, and between compliance and independent variables were computed using IBM SPSS Statistics 25. OLS regression analyses with robust standard errors (Huber-White sandwich estimator) were conducted using STATA 16.0.

#### Network Analysis

Network analysis was conducted in R (R Core Team, 2013) with the package *mgm* (Haslbeck & Waldorp, 2020) with all pairwise interactions (*k* = 2). Mgm (Mixed Graphical Models) is used to estimate a network from different types of data, such as continuous variables (e.g., compliance) and categorical variables (e.g., knowledge of measures). The network was estimated using nodewise regression. Edge selection was based on tenfold cross-validation, and an edge was included in the network if any of the two possible directions between edges were selected. The clusters in the networks were determined through cluster stability and detection analysis with a cluster walktrap algorithm (see Online Appendix C). The R code for the network analysis can be found in Online Appendix C.

We assess the centrality of nodes, which indicate their position in the network. Centrality is assessed by calculating the *node strength*, i.e., the sum of the strength of (absolute) associations (edge weights) to connected nodes (Epskamp et al., 2018a, b, c). The bootstrapped centrality difference test (Epskamp et al., 2018a) is conducted to test for significant differences between nodes. The R code or the network analysis can be found in Online Appendix C.

## Results

### Compliance

Table 4 shows the descriptive statistics of the compliance measures. For all items, participants reported relatively high levels of compliance. Responses to all five items were mean-scored to create a combined scale measure of compliance (see “Appendix A” for more details).

**Table 4:** Descriptive statistics for compliance measures

Item	Mean	SD	Scale
*Since the authorities took measures to contain the Coronavirus:*			
Social distancing			
I still meet people outside of my direct household^a^	5.89	1.10	1–7
I keep a safe distance from people outside of my direct household	6.04	1.16	1–7
I still visit others (friends, relatives) outside of my direct household^a^	6.24	0.97	1–7
I still allow others (friends, relative) to visit my direct household^a^	6.16	0.97	1–7
Stay at home			
I have stayed at home after I was ordered to do so, apart from engaging in essential activities (e.g., grocery shopping, medical appointments)	5.93	1.24	1–7
Compliance (combined scale measure)	6.05	0.72	1–7

^a^Reverse scored

### Descriptive Statistics

Table 5 shows the descriptive statistics for the independent and additional variables.

**Table 5:** Descriptive statistics M(SD) of variables

Variables	M(SD)	Scale
Rational choice theories		
Costs of compliance	4.04 (1.35)	1–7
Perceived threat	4.97 (1.10)	1–7
Punishment certainty	3.48 (1.27)	1–7
Punishment severity	3.61 (1.15)	1–6
Social theories		
Social norms	5.40 (1.15)	1–7
Legitimacy theories		
Moral support	6.25 (1.06)	1–7
Support for policies	4.47 (1.19)	1–7
Obligation to obey the law	4.35 (1.70)	1–7
Procedural justice	5.36 (1.09)	1–7
Capacity theories		
Capacity to comply	5.40 (1.13)	1–7
Knowledge of measures	5.21 (1.67)	0–7
Clarity of measures	5.31 (1.32)	1–7
Impulsivity	2.11 (0.80)	1–5
Negative emotions	4.08 (1.16)	1–7
Opportunity theories		
Opportunity to violate	3.84 (1.38)	1–7

### Correlations and Regression Analysis

“Appendix B” displays the results of the correlational analyses. These revealed significant correlations between compliance and variables from several of the major theoretical approaches: benefits of complying, in terms of perceived threat (rational choice theories), social norms (social theories), moral support, obligation to obey the law, and procedural justice (legitimacy theories), capacity to comply, knowledge of measures, clarity of measures, impulsivity, and negative emotions (capacity theories), and opportunity to violate (opportunity theories). As such, this suggested that all five of the major theoretical approaches were associated with individual compliance in this setting—although perceived costs of compliance, punishment certainty, and punishment severity (rational choice theories) were unrelated to compliance.^3^

Next, we estimated a regression model (with robust standard errors) in which the independent variables (i.e., the compliance mechanisms) were entered as independent variables, and the compliance scale measure as dependent variable. The analysis controlled for all additional variables that displayed significant correlations with compliance. Collinearity statistics showed no issues with multicollinearity (VIF ≤ 1.36, tolerances ≥ 0.73). The results are displayed in Table 6. Compliance was significantly predicted by perceived threat (rational choice theories), moral support for measures (legitimacy theories), practical capacity to comply, knowledge of measures, and impulsivity (capacity theories), and opportunity to violate (opportunity theories).^4^ As such, when considering their unique contributions, four of the major compliance theories contributed variables that showed significant associations with individual compliance. However, several other variables from these theoretical families no longer showed significant associations with compliance (i.e., obligation to obey the law, procedural justice, clarity of measures, and negative emotions), while social theories no longer predicted this outcome.

**Table 6:** Linear regression of compliance (with robust standard errors), adjusted for control variables

	*B*	SE	Effect size (Cohen’s d)
Additional variables			
Age	0.00	0.00	0.07
Gender	0.12*	0.06	0.18
Household size	− 0.02	0.02	0.11
Health self	0.09	0.07	0.10
Health other	0.07	0.06	0.10
Trust in science	0.07	0.04	0.17
Rational choice theories			
Costs of compliance	0.04	0.02	0.16
Perceived threat	0.07*	0.03	0.21
Punishment certainty	− 0.03	0.02	0.12
Punishment severity	0.04	0.02	0.14
Social theories			
Social norms	0.05	0.02	0.18
Legitimacy theories			
Moral support	0.13***	0.04	0.39
Support for policies	− 0.05	0.03	0.17
Obligation to obey the law	0.02	0.02	0.08
Procedural justice	− 0.00	0.03	0.01
Capacity theories			
Capacity to comply	0.09***	0.03	0.32
Knowledge of measures	0.08***	0.02	0.43
Clarity of measures	0.02	0.02	0.08
Impulsivity	− 0.12**	0.04	0.31
Negative emotions	− 0.00	0.03	0.02
Opportunity theories			
Opportunity to violate	− 0.06*	0.02	0.23
R^2^	0.30		

### Network of Factors Related to Compliance

**Fig. 1:**
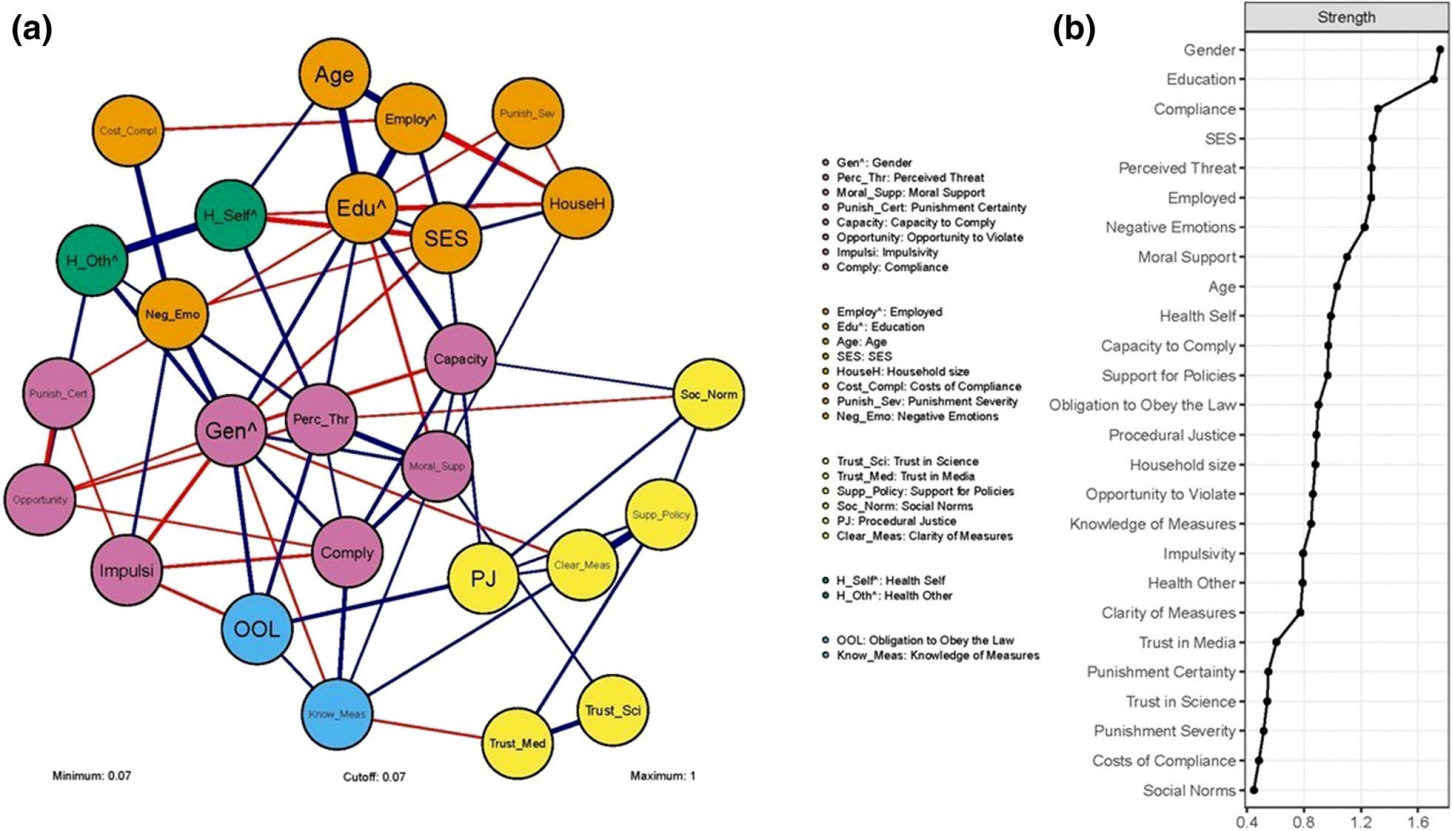
**a** Psychological network of factors related to compliance with COVID-19 mitigation measures. Nodes represent measured compliance mechanisms and edges represent relations between nodes (blue = positive, red = negative). Positive relations with binary nodes (marked ^) indicate that increasing the other node results in a higher probability for category one of the binary node (i.e. Gender 1 = Female; Education 1 = Higher; Employed 1 = Yes, Health Self 1 = Yes; Health Other 1 = Yes). Edge width and color density indicate the strength of relations (edge weight). Edges with weights below a value of 0.07 are omitted; **b** centrality measure ‘strength’ for each node in the network. This measure represents the average conditional association between that node and other nodes in the network, and is calculated by the sum of the absolute edge weights of the relations a specific node has with connected nodes. (Color figure online)

Figure 1a shows the network of factors related to individual compliance in this setting. In this network, the nodes (the circles) represent the variables originating from the different compliance theories (as well as the control variables), and the edges (the lines) the relationships between them. The edges are undirected: this means that the nodes connected by the edge have some mutual relationship, but any causal direction of this relationship is undetermined. Furthermore, the edges are weighted: this means that the thickness of the edge reflects the strength of the relationships between the nodes (Haslbeck & Waldorp, 2020). The edge may reflect a positive relation (blue edges), or a negative relation (red edges). Edge weights with values below 0.07 are omitted in the visualization of the network.^5^

The colored groups are clusters: groups of nodes that show higher connectedness with nodes within that cluster than with nodes outside of it (Borsboom et al., 2011). In this network, we can distinguish five clusters. Table 7 presents the clusters as expected based on the five major theoretical families (columns 1–2), and the clusters as observed in the data (columns 3–7). As can be seen, the observed clusters in the network did not match the expected clusters. Rather, every cluster contained factors from multiple theories, and none of the clusters lined up with one specific theory. The purple cluster represents the cluster of nodes related to compliance. It consists of nodes belonging to rational choice theories (*Perceived Threat* and *Punishment Certainty*), legitimacy theories (*Moral Support*), capacity theories (*Capacity to Comply* and *Impulsivity*) and opportunity theories (*Opportunity to Violate*). As such, it encompasses variables from four different theoretical families, which showed strong interconnections with each other, but weak connections with other variables from their respective theoretical families. It suggests that in the present empirical setting, perceptions of the rules (such as one’s support and their perceived benefits) were closely aligned with perceptions of their practical feasibility (i.e., one’s perceived capacity to comply, opportunities for offending, and perceived consequences of doing so). This implies that more favorable perceptions of rules may coincide with greater perceived practical feasibility of following them.

**Table 7:** Overview of variables that were expected to cluster based on theories and observed clusters

Theory	Variable/node	Cluster 1 (purple)	Cluster 2 (yellow)	Cluster 3 (orange)	Cluster 4 (blue)	Cluster 5 (green)
	Compliance	x				
Rational choice	Perceived threat	x				
	Punishment certainty	x				
	Punishment severity			x		
	Costs of compliance			x		
Social	Social norms		x			
Legitimacy	Moral support	x				
	Support for policies		x			
	Procedural justice		x			
	Obligation to obey the law				x	
Capacity	Capacity to comply	x				
	Impulsivity	x				
	Clarity of measures		x			
	Negative emotions			x		
	Knowledge of measures				x	
Opportunity	Opportunity to violate	x				
Additional variables	Trust in science		x			
	Trust in media		x			
	Gender	x				
	Employed			x		
	Education			x		
	Health self					x
	Health other					x
	Age			x		
	SES			x		
	Household size			x		

The yellow cluster, containing the perceived social norm surrounding compliance (*Social Norms*), indicates interconnectedness of nodes from legitimacy theories (*Support for Policies* and *Procedural Justice*), capacity theories (*Clarity of Measures*) and (supplementary) variables concerning trust (*Trust in Science* and *Trust in Media*). This suggests that people’s perceptions of the legitimacy of authorities may be closely aligned with the clarity of their measures, and the extent to which others are seen to comply with them. As such, greater perceived legitimacy of authorities may coincide with stronger perceived norms for following their measures.

The orange cluster, containing most demographic variables, reveals interconnectedness with variables from rational choice theories (*Punishment Severity* and *Costs of Compliance*) and capacity theories (*Negative Emotions*). Hence, negative consequences here seemed to be interconnected with negative emotions, or alternatively, people’s perceptions of such consequences were connected to their emotional state.

The blue cluster suggests higher connectedness between variables from legitimacy theories (*Obligation to Obey the Law*) and capacity theories (*Knowledge of Measures*)*.* This suggests that greater perceived legitimacy of authorities may coincide with greater practical knowledge of the rules they make. In sum, these findings show important interrelations between the variables that are obscured in traditional, non-network approaches to individual compliance—moreover, they thereby reveal associations that do not align with the major theoretical families in compliance research.

Table 8 displays the edge weights corresponding with the compliance network, indicating the strength of the relationship between each pair of nodes (variables). As can be seen, the analysis revealed positive associations (edges) between *Compliance* and several of the theorized variables, the strongest of which were *Knowledge of Measures* (0.19) and *Capacity to Comply* (0.15; capacity theories), *Moral Support* (0.18; legitimacy theories), and *Perceived Threat* (0.10; rational choice theories).,^6^^,^^7^ Additionally, *Compliance* had negative relations with *Impulsivity* (− 0.12; capacity theories), *Opportunity to Violate* (− 0.07; opportunity theories) and *Support for policies* (− 0.07, rounded up; legitimacy theories).

**Table 8:** Edge weights of the compliance network. This table includes the weights of all edges in the network, including those with edge weights below 0.07 that are omitted from Fig. 1. Information on the edge accuracy is provided in Online Appendix C

	Gender	Employed	Education	Health Self	Health Other	Age	SES	Household size	Trust in Science	Trust in Media	Perceived Threat	Moral Support	Support for Policies
Gender			0.16		0.18		− 0.11					0.13	
Employed			0.35			0.34	0.20	− 0.12					− 0.07
Education						0.36	0.12	− 0.14			0.13	− 0.11	
Health Self					0.39	0.12	− 0.22	− 0.09			0.17		
Health Other													
Age								− 0.10					
SES								0.13					0.05
Household size												0.08	0.04
Trust in Science										0.22		0.08	0.04
Trust in Media											0.06		0.13
Perceived Threat												0.25	
Moral Support													
Support for Policies													
Costs of Compliance													
Punishment Certainty													
Punishment Severity													
Capacity to Comply													
Opportunity to Violate													
Social Norms													
Impulsivity													
Obligation to Obey the Law													
Procedural Justice													
Knowledge of Measures													
Clarity of Measures													
Negative Emotions													
Compliance													

	Costs of Compliance	Punishment Certainty	Punishment Severity	Capacity to Comply	Opportunity to Violate	Social Norms	Impulsivity	Obligation to Obey the Law	Procedural Justice	Knowledge of Measures	Clarity of Measures	Negative Emotions	Compliance
Gender		− 0.06		− 0.14	− 0.08		− 0.16	0.19		− 0.08	− 0.08	0.26	0.13
Employed	− 0.11						0.05		0.04				
Education		− 0.06		0.22								0.06	
Health Self													
Health Other					0.13							0.08	
Age	− 0.06											− 0.06	
SES			0.19	0.09					0.10			− 0.07	
Household size			− 0.08									− 0.05	− 0.05
Trust in Science								0.07	0.07				0.06
Trust in Media						0.05			0.06	− 0.09			
Perceived Threat			− 0.04		− 0.09	− 0.07		0.16		0.05		0.14	0.10
Moral Support				0.10					0.04	0.09	0.04		0.18
Support for Policies					0.04	0.09			0.09		0.36		− 0.07
Costs of Compliance				− 0.03								0.24	0.05
Punishment Certainty			− 0.08		− 0.18		− 0.07			0.03	0.07		
Punishment Severity							0.03					− 0.04	0.05
Capacity to Comply					0.05	0.08			0.08	0.03			0.15
Opportunity to Violate							− 0.07	− 0.04		− 0.03	− 0.03	− 0.05	− 0.07
Social Norms									0.12				0.05
Impulsivity								− 0.11		0.04		0.13	− 0.12
Obligation to Obey the Law									0.18	0.10			0.04
Procedural Justice											0.09	− 0.04	
Knowledge of Measures											0.11		0.19
Clarity of Measures													
Negative Emotions													
Compliance													

However, beside the relations with *Compliance*, the network also revealed numerous interrelations among the theorized variables, including between variables originating from different theoretical families. For example, *Perceived Threat* (rational choice theories) was also directly associated with greater *Moral Support* (0.25) and *Obligation to Obey the Law* (0.16; legitimacy theories).In addition, salient was the strong positive relation between *Support for Policies* (legitimacy theories) and *Clarity of Measures* (0.36; capacity theories). In sum, the network analysis confirmed several of the associations with compliance that had been observed in the correlational and regression analyses. Importantly, however, it also revealed sizable interrelations that suggest these variables may indirectly influence each other and compliance, and also be (directly and indirectly) influenced by compliance. Moreover, many of these associations involve variables originating from different theoretical families.

#### Centrality

Lastly, we examined the centrality of the nodes by calculating the *node strength*. Simply put, nodes that have more, and/or stronger associations with other nodes will have a higher node strength, indicating greater relative importance for the overall network. Figure 1b displays these values, and Online Appendix C shows the results from the centrality difference test. The centrality difference test did not find significant differences between the theoretical variables nor a clear hierarchical structure. Therefore, centrality analysis did not single out any particular theory as being especially influential. Rather, most theoretical variables were broadly comparable in terms of node strength, suggesting them to have comparable effects on (and to be comparably affected by) the other variables in the network.^8^ As with the clusters, node strength did not reveal a pattern that aligned with the major theoretical families in compliance research.

## Discussion

Understanding the processes that shape individual compliance is essential for the study of corporate compliance. The goal of the present research was to showcase a network approach to individual compliance, where it studied individual compliance as part of a complex system of variables originating from the key compliance theories from the social and behavioral sciences. It did so in the setting of behavioral responses to COVID-19 mitigation measures during the first pandemic wave, in a sample from the Netherlands. We aimed to demonstrate how a network approach to individual compliance can advance our understanding beyond these siloed theoretical families, and beyond traditional, non-network analyses. We thereby tried to illustrate how this approach can be utilized to deepen our understanding of individual compliance in other settings in corporate or everyday life.

Results showed that individual compliance within this setting was shaped by a combination of mechanisms, originating from rational choice theories (i.e., perceived threat, Donovan & Blake, 1992; Paternoster & Simpson, 1993), legitimacy theories (i.e., moral support for the measures; Tyler, 1997, 2006), capacity theories (i.e., capacity to comply, Darley et al., 2001; knowledge of the rules, Van Rooij, 2021; self-control, Gottfredson & Hirschi, 1990), and opportunity theories (i.e., opportunities for violating the rules, Clarke, 2003, 2005). A first noteworthy observation from our approach is therefore that in this setting, most of the major compliance theories (with the exception of social theories) offered variables that were associated with individual compliance, while none of the theories exclusively explained it. Although further research is needed to understand how these processes may operate in other empirical settings, these findings do suggest that attempts to understand individual compliance from a singular theoretical perspective are likely to overlook critical aspects of this question. Instead, the present findings suggest that understanding individual compliance requires a multi-theoretical perspective.

The results from the network approach showed that the clusters of variables in the network (i.e., variables that show higher connectedness with each other than with other variables, as indicated by the colored groups in Fig. 1a) did not at all cluster along the expected theoretical lines (apart from theories represented by only a single variable, such as social theories and opportunity theories). Contrary to their siloed theoretical families, the observed clusters consisted of variables from multiple theories, and variables originating from the same theory often were spread into different clusters in the network. This was even the case for variables that theoretically seem closely aligned, such as certainty and severity of punishment. The network approach to individual compliance therefore showed that, at least in the empirical setting studied here, existing theories do not provide sufficient conceptual basis to separate different distinct and coherent perspectives of how individual compliance takes place in reality. Rather, the empirical network shows that compliance does not follow existing distinct theoretical patterns.

Furthermore, the results from the network approach showed the interrelations between variables from different bodies of theories, beside their associations with compliance. In more traditional statistical approaches that regard them as independent or interacting variables without looking at the full network of interactions that exist, this is obscured. The network revealed noteworthy associations between all the theoretical compliance variables and compliance itself, both directly and indirectly. It shows that in this empirical setting, the variables do not operate independently but rather interact in a complex network. For example, the network showed that variables that were directly connected to compliance (perceived threat, moral support, capacity to comply, knowledge of the measures, impulsivity, and opportunity to violate) were in turn widely connected to other variables from throughout the network, which thus may influence (and be influenced by) them. Thus, a network approach to compliance could help to expose possible indirect ways in which the variables may influence each other, including compliance. Insight into such structures of direct and indirect connections between variables may improve our understanding of interventions. That is, effects of interventions aimed at improving compliance can be better understood with insight into connected variables and their tendency to align or not. For instance, reinforcing triangular motifs (i.e., positive relations between three connected variables) can amplify intervention effects: increasing the first node can lead to increasing the second node, which can lead to increasing the third node, in turn further increasing the first node. Conversely, attenuating triangular motifs (e.g., negative relations between three connected variables) might weaken the effects of interventions due to conflicting effects. Optimizing our understanding of such motifs requires insight into directions of relations between variables, which future research with repeated measures, or more advanced methods which simulate interventions, could obtain.

### Implications

The findings have several implications for the practice, study, and theory of compliance. Most specifically, to begin with, our study contributes to the literature on compliance with COVID-19 mitigation measures. Although our research primarily utilized this setting as a suitable context for developing our network approach to individual compliance, our findings do connect with other studies that specifically aim to understand mitigation behaviors by drawing from the major compliance theories (for a review, see Kooistra & Van Rooij, 2020; also, see Reinders Folmer et al., 2021). Importantly, our network approach suggests complex interrelations between many of the variables that have been associated with compliance in this setting—interrelations which have not been considered in the traditional, non-network approaches utilized in prior COVID-19 compliance research. Although it is not yet clear if the observed associations will extend to other samples and populations, these observations do suggest that also there, our understanding of COVID-19 compliance may be considerably enriched by adopting a network approach to individual compliance.

More broadly our study has implications for compliance theory and practice outside of the realm of COVID-19. Here, our findings fundamentally challenge the existing theoretical approaches to compliance as proposed in the major families of compliance research from across the social and behavioral sciences. Individual compliance in the present study comprises an interplay of variables that defies existing theoretical categorization. While the observed network in itself may not generalize to other samples or settings, this finding does raise important questions about the tenability of such narrow conceptualizations of compliance. As noted previously, other compliance research also provides indications that different influences on compliance may not operate independently from each other and from compliance (e.g., Bar-Gill & Harel, 2001; Cialdini et al., 2006; Gneezy et al., 2011). On the one hand, this may signal a need for novel, integrative theories based on such observed associations. On the other hand, a network perspective implies that individual compliance may be best understood as part of an interrelated network of variables, which are associated with each other in complex ways and thus may shape compliance through multiple pathways. This demands a new view on compliance, rooted in complexity science, which moves beyond narrow conceptions of this complex phenomenon.

The present study also has methodological implications for future compliance research. By demonstrating how individual compliance in the present empirical setting can be understood by means of network analysis that integrates the key theoretical approaches to compliance, our research provides a template with which compliance can be studied in other empirical settings. Such studies should strive to understand individual compliance by mapping relevant concepts from across the major theories: incentives, norms, and legitimacy perceptions, but also people’s capacity for following the rules and situational opportunities. This may be expanded with other relevant influences that operate within particular settings (e.g., compliance management systems, culture, leadership). Their associations to compliance should be assessed through a complex systems approach, using for example network analysis. This will illuminate the complex network of factors that interact with compliance within this particular setting. Moreover, understanding such networks may help to better understand the impact of compliance interventions, and to uncover novel ways of doing so. Network analysis can show how simulated, targeted interventions may affect a whole range of other variables throughout the network, and help to predict (direct or indirect, positive or negative) associations with compliance (Lunansky et al., 2021). The network approach to compliance moves beyond the assumption that different compliance variables operate independently or through simple interactions. Instead, it can show the complex associations between all relevant variables and the way that these come to interact with compliance. This points the way to the next frontier in individual and corporate compliance research, using complexity science to start modeling compliance interventions to assess their effects within the network (e.g., see Henry et al., 2021; Lunansky et al., 2021; Robinaugh et al., 2016).

These broad implications for compliance theory are also essential for (corporate) compliance practice. The findings about individual compliance hold direct relevance for those studying corporate compliance, as corporate compliance scholarship uses the same theoretical and methodological approaches and has not yet developed an integrated view of how compliance variables interrelate with each other and compliance. Our study shows that reliance on interventions that follow singular theoretical approaches could come with a high risk of ineffectiveness, and at worst unintended negative consequences. Thus, a network approach to compliance can form the basis for improving compliance management programs and regulatory interventions.

### Limitations

Some limitations of this study should be recognized. As a first limitation, we used self-reported compliance data that may be subjected to response biases, such as imperfect recall or social desirability bias (Bauhoff, 2011; Hansen et al., 2021; Van de Mortel, 2008). However, the finding of high self-reported compliance is in line with objective mobility data from the same period (Google, 2020). Also, existing research shows that there can be strong concordance between self-reported and objective compliance measures when surveys are utilized (Bachmann et al., 1999; Dieltjens et al., 2013; Garber et al., 2004; Rauscher et al., 1993; Ridgers et al., 2012). Furthermore, a recent study found that social desirability bias did not inflate the estimates of compliance with COVID-19 measures in online surveys (Larsen et al., 2020).

Second, our study did not include a representative sample, and thus is not suitable for inferences about specific populations (such as that of the Netherlands), or for policy recommendations. It should be noted, however, that this was not the purpose of our research. Rather, its purpose was theoretical and methodological: to understand individual compliance as part of a complex system of interrelated variables, and to employ a network approach that can be used to study this in other (corporate and everyday) settings. This objective does not require a nationally representative sample; rather, what it requires is high-quality data: participants who pay attention, understand instructions, provide truthful responses, and provide internally consistent responses. Research has demonstrated that online crowdsourcing platforms are mostly comparable to university and community samples in these regards (Behrend et al., 2011; Goodman et al., 2013), and that especially Prolific (through which our study was conducted) outperforms other crowdsourcing platforms and research panels (Peer et al., 2017, 2021). Accordingly, our sample was suitable for our theoretical and methodological purposes, but should not be utilized to make inferences about other populations or policies. This also means, as previously noted, that relationships observed within the present empirical setting should not be directly generalized to other (corporate and everyday) settings. Rather, the major recommendation of our work is that the network approach that we demonstrate here should be utilized to understand compliance in those settings.

The network analysis explores relations between variables, and as such, we cannot make claims about the causality of relationships. Further research is needed to understand the direction of the relationships between different compliance mechanisms within our network. Given that the causal relationship of compliance is hard to study in practice (van Rooij & Sokol, 2021), experimental approaches that zoom in on specific associations could be utilized to disentangle this. Additionally, longitudinal data could shed some light on how associations in compliance networks may develop over time. Networks of individual participants that are followed over a period of time may show unidirectional associations between compliance and related variables, thereby potentially unraveling mechanisms that influence compliance behavior (see Bringmann et al., 2013; Epskamp et al., 2018b for methods to estimate longitudinal networks).

### Conclusion

The results of this study point to a new complexity science research agenda for compliance research, using network analysis. By studying the compliance networks that occur in other settings in everyday and corporate life, such research first can help to illuminate the network of factors that shape compliance in those settings. Moreover, comparison of compliance networks (for example, using the statistical Network Comparison Test, Van Borkulo et al., 2022) may also help to understand how compliance networks may show stability or variability across different settings: how differences between settings, such as between different companies, sectors, countries, or regulatory frameworks, may be reflected in the way that particular concepts may relate to each other in the network. Such insight will be of great importance for the development of novel theories, which take into account the complex relationships between these concepts, and the way in which these may vary depending on their context. In this manner we can go beyond the tunnel vision that obscures a fuller understanding of the empirical reality of how different variables come to shape compliance.

### Electronic supplementary material

Below is the link to the electronic supplementary material.

Supplementary file1 (DOCX 573 kb)

## Appendix A

### Materials

For a complete overview of all survey materials and items, please see https://osf.io/3x5cq/. Below, we provide a global description of the variables included in this study. Unless otherwise specified, mean scores were calculated for all variables included.

#### Additional Variables

The following descriptive statistics were recorded: age, gender, employment status, education, social economic status before COVID-19 (MacArthur Scale of Subjective Social Status; Adler et al., 2000). Furthermore, participants indicated whether they themselves or anyone they knew had underlying health issues that would make them more at-risk for contracting COVID-19. Finally, participants were asked to indicate their trust in science (on a single item taken from McCright et al., 2013), and trust in media reporting on a single item, similar to the item of trust in science.

#### Compliance with COVID-19 Measures

To assess compliance, we focused on two main COVID-19 mitigation measures: “social distancing” and “stay at home” measures. Four items (*α* = 0.65) measured whether participants complied with social distancing measures. For social distancing, three items were reverse scored. Compliance with stay-at-home measures was measured using a single item. Participants answered on a 7-point Likert scale ranging from (1) “never” to (7) “always.” A factor analysis of the compliance measures resulted in all items loading on one factor; moreover, the reliability of a combined scale including both social distancing and stay at home measures (*α* = 0.68) exceeded that of either measure individually. For these reasons, all five items were combined into a scale measure, with higher values indicating greater compliance with COVID-19 mitigation measures.

#### Rational Choice Theories

The mechanisms measured in line with rational choice theories were the costs of compliance, perceived threat of the virus, and deterrence. Deterrence was measured on two subscales: certainty of punishment and severity of punishment.

##### Costs of Compliance

Participants indicated on five items (*α* = 0.74) how likely it was that compliance with the COVID-19 mitigation measures would have a negative impact on them (e.g., loss of work, income, or social contacts). Participants answered on a 7-point Likert scale ranging from (1) “extremely unlikely” to (7) “extremely likely.” A higher score indicated that people thought it was more likely that COVID-19 measures would have a negative impact on them.

##### Perceived Threat

Perceived threat was measured using three items (*α* = 0.76), on which participants indicated to what extent they believed the Coronavirus was a threat to themselves, friends and relatives, or the general public, rated on a 7-point Likert scale ranging from (1) “very strongly disagree” to (7) “very strongly agree.” Higher scores indicated higher perceived threat.

##### Certainty of Punishment

Two items each measured the perceived certainty of apprehension and punishment for violating social distancing measures (*α* = 0.72) and stay at home measures (*α* = 0.81), answered on a 7-point Likert scale ranging from (1) “extremely improbable” to (7) “extremely probable.” Because perceptions for social distancing and stay at home measures were highly correlated, they were combined into an aggregated scale measure of certainty of punishment (*α* = 0.80). Higher scores indicated greater certainty of punishment for violating COVID-19 measures.

##### Severity of Punishment

One item each assessed the perceived severity of punishment for violating social distancing measures and stay at home measures. Specifically, participants indicated how much they believed they would suffer if punished for violating these measures, on a 6-point Likert scale ranging from (1) “extreme suffering” to (6) “no suffering at all,” reverse coded. Again, the measures for social distancing measures and stay at home measures were highly correlated, and thus were combined into an aggregated measure (*r* = 0.72, *p* < 0.001), with higher scores indicating higher perceived severity of punishment for violating COVID-19 measures.

#### Social Theories

The mechanism measured in line with social theories was descriptive social norms.

##### Social Norms

Participants rated to what extent people they know comply with the COVID-19 measures (one item for each measure, *α* = 0.87), on a 7-point Likert scale ranging from (1) “very strongly disagree” to (7) “very strongly agree.” Higher scores indicated more compliant descriptive social norms.

#### Legitimacy Theories

The mechanisms measured in line with the legitimacy theories were substantive moral support, including specific moral alignment and support for current policies, procedural justice, and obligation to obey the law.

##### Moral Support

Moral support for measures was measured using two items (*r* = 0.54, *p* < 0.001), on which participants indicated to what extent they believed people should follow the COVID-19 mitigation measures, rated on a 7-point Likert scale ranging from (1) “very strongly disagree” to (7) “very strongly agree.” Higher values indicated more specific moral alignment.

##### Support for Policies

Support for current policies was measured using three items, on which participants indicated to what extent they supported the authorities in adopting the COVID-19 mitigation measures, rated on a 7-point Likert scale ranging from (1) “very strongly disagree” to (7) “very strongly agree.” Scale reliability was low (*α* = 0.40) as one item displayed a poor item-total correlation; the remaining two items were strongly correlated, however (*r* = 0.62, *p* < 0.001). As such, these were combined into a scale measure, with higher values indicating more support for the authorities adopting the COVID-19 measures.

##### Obligation to Obey the Law

Obligation to obey the law was measured with a single item, “I feel like it is sometimes okay to break the law”, on a 7-point Likert scale ranging from (1) “strongly agree” to (7) “strongly disagree.” This item was created for the current study based on existent work (e.g., Estévez & Emler, 2010; Fine et al., 2020; Reisig et al., 2007). Higher scores indicated greater obligation to obey the law.

##### Procedural Justice

Procedural justice was measured by adapting instruments for evaluating perceived fairness of law enforcement (Baker & Gau, 2018; Gau, 2014; Tyler, 1997; Wolfe et al., 2016). Three items measured procedural justice in creating the COVID-19 mitigation measures, and four items measured procedural justice in enforcing the COVID-19 mitigation measures. The items were answered on a 7-point Likert scare ranging from (1) “very strongly disagree” to (7) “very strongly agree.” Because the procedural justice perceptions for creation and enforcement were strongly correlated, all seven items were combined into a scale measure of perceived procedural justice (*α* = 0.91), with higher values indicating higher procedural justice.

#### Capacity Theories

The measures of the theories on capacity to comply were practical capacity to comply, knowledge of current measures, perceived clarity of current measures, impulsivity, and negative emotions.

##### Capacity to Comply

Three items (*α* = 0.53) measured to what extent participants are practically able to comply with the COVID-19 mitigation measures, on a 7-point Likert scale ranging from (1) “very strongly disagree” to (7) “very strongly agree.” Higher scores indicated more practical ability to comply.

##### Knowledge of Measures

Participants were presented with seven items to assess their knowledge of the COVID-19 mitigation measures that currently applied to them (1 = yes, 2 = no, 3 = unsure). As all measures mentioned in these items were effectively in force within the Netherlands at time of the survey, the number of correct answers (1 = yes) was counted, and used as an indicator of knowledge of current measures, with higher scores indicating greater knowledge.

##### Clarity of Measures

Furthermore, participants were asked on one item whether the COVID-19 measures were clear to them, answered on a 7-point Likert scale ranging from (1) “extremely unclear” to (7) “extremely clear.” Higher scores indicated greater clarity of the current measures.

##### Impulsivity

Impulsivity was measured using a subset of five items (*α* = 0.76) taken from the 8-item impulse control subscale from the Weinberger Adjustment Inventory (WAI; Weinberger & Schwartz, 1990). The items were answered on a 5-point Likert scale ranging from (1) “false” to (5) “true.” One item was reverse scored, and higher scores indicated higher impulsivity.

##### Negative Emotions

Negative emotions due to COVID-19 was measured on six items (*α* = 0.84) assessing different negative emotions, rated on a 7-point Likert scale ranging from (1) “very strongly disagree” tot (7) “very strongly agree.” Higher values indicated higher negative emotions.

#### Opportunity Theories

The mechanism in line with opportunity theories was opportunity to violate.

##### Opportunity to Violate

Five items (*α* = 0.78) measured to what extent participants had the opportunity in practice to violate the COVID-19 mitigation measures, on a 7-point Likert scale ranging from (1) “very strongly disagree” to (7) “very strongly agree.” Higher scores indicated more opportunity to violate the COVID-19 mitigation measures.

## Appendix B

See Tables 9 and 10.

**Table 9:** Kendall’s tau correlations of control variables (*N* = 562)

	Age	Gender	Education	Employed	Household size	Ethnicity	SES	Health self	Health other	Trust in science	Trust in media
Age											
Gender	0.03										
Education	0.42**	0.09*									
Employed	0.37**	0.00	0.30**								
Household size	− 0.25**	− 0.08*	− 0.21**	− 0.15**							
Ethnicity	0.10**	− 0.11*	0.01	0.06	− 0.01						
SES	0.03	− 0.13**	0.12**	0.15**	0.09**	0.11**					
Health self	0.07	00.07	0.03	0.04	− 0.11**	− 0.03	− 0.17**				
Health other	0.06	0.13**	0.10**	0.06	− 0.02	0.06	0.03	0.14**			
Trust in science	− 0.03	− 0.04	0.07	− 0.04	− 0.01	0.10*	0.06	− 0.01	0.07		
Trust in media	0.07*	− 0.02	0.07*	0.03	− 0.04	0.09*	0.08*	0.02	0.06	0.26**	
Compliance	0.08*	0.13**	0.04	0.00	− 0.10**	− 0.06	− 0.02	0.09*	0.08*	0.09**	0.03

Ethnicity—*N* = 551

*Correlation is significant at the 0.05 level

**Correlation is significant at the 0.01 level

**Table 10:** Kendall’s tau correlations of independent variables (*N* = 562)

	Costs of compliance	Perceived threat	Certainty of punishment	Severity of punishment	Social norms	Moral support	Support for policies	Obligation to obey the law	Procedural justice	Capacity to comply	Knowledge of measures	Clarity of measures	Impulsivity	Negative emotions	Opportunity to violate
Costs of compliance															
Perceived threat	0.08**														
Certainty of punishment	0.05	0.07*													
Severity of punishment	− 0.03	− 0.06	− 0.08*												
Social norms	− 0.08*	− 0.02	0.02	0.01											
Moral support	0.01	0.36**	0.02	0.06	0.08*										
Support for policies	− 0.05	0.01	0.08*	− 0.02	0.13**	0.00									
Obligation to obey the law	0.01	0.22**	0.06*	− 0.00	0.06	0.17**	0.07*								
Procedural justice	− 0.03	0.04	0.04	0.00	0.16**	0.11**	16**	0.19**							
Capacity to comply	− 0.07*	0.10**	0.00	0.06	0.15**	0.26**	0.05	0.10**	0.14**						
Knowledge of measures	0.04	0.17**	0.07*	− 0.01	0.02	0.20**	− 0.01	0.16**	0.06	0.12**					
Clarity of measures	− 0.04	0.09**	0.12**	0.04	0.13**	0.12**	0.34**	0.13**	0.18**	0.09**	0.14**				
Impulsivity	0.00	0.01	− 0.06	0.04	− 0.00	− 0.01	− 0.00	− 0.14**	− 0.04	− 0.05	0.04	− 0.04			
Negative emotions	0.21**	0.18**	0.02	− 0.07*	− 0.06	0.05	− 0.08*	0.08*	− 0.07*	0.02	0.05	− 0.06	0.09**		
Opportunity to violate	− 0.02	− 0.15**	− 0.16**	0.02	0.02	− 0.09**	0.03	− 0.10**	− 0.06	0.02	− 0.11**	− 0.07*	− 0.06	− 08**	
Compliance	0.04	0.23**	− 0.01	0.05	0.08*	0.37**	− 0.05	0.20**	0.09**	0.23**	0.24**	0.08*	− 0.10**	0.06*	− 0.08*

*Correlation is significant at the 0.05 level

**Correlation is significant at the 0.01 level

## Footnotes

1 The Netherlands is ranked first regarding English skills after exclusion of native English countries (https://www.ef.com/wwen/epi); more than 90% of Dutch people rate themselves as reasonable to fluent in English (Edwards, 2014).

2 One participant was excluded based on overlap in exclusion criteria: both providing professional care and failing the attention check.

3 Additionally, these analyses revealed significant associations between compliance and several supplementary variables: age, gender, household size, socio-economic status, health self, health other, and trust in science.

4 Of the control variables, gender significantly predicted compliance, such that compliance was greater among women.

5 More information on the accuracy of edge weights and formal edge difference tests is provided in Online Appendix C.

6 Edge difference tests (see Online Appendix C) indicated that these relations with Compliance were of comparable strength, meaning that these did not differ significantly.

7 Please note that the score on the node *Knowledge of Measures* leaned toward a bimodal distribution, which could potentially lead to instable results. The edge accuracy analysis (see Online Appendix C) provides information to evaluate the accuracy of the edges with *Knowledge of Measures*.

8 More information about the stability of this measure for the network of factors related to compliance and the results from the formal node strength difference tests can be found in Online Appendix C.

## References

[CR1] Adler NE, Epel ES, Castellazzo G, Ickovics JR (2000). Relationship of subjective and objective social status with psychological and physiological functioning: Preliminary data in healthy, White Women. Health Psychology.

[CR2] Agnew R (1992). Foundation for a general strain theory of crime and delinquency. Criminology.

[CR3] Agnew R (2007). Pressured into crime: An overview of general strain theory.

[CR4] Agnew R, White HR (1992). An empirical test of general strain theory. Criminology.

[CR5] Agnew R, Brezina T, Wright JP, Cullen FT (2002). Strain, personality traits, and delinquency: Extending general strain theory. Criminology.

[CR6] Akers RL, Jensen GF (2011). Social learning theory and the explanation of crime.

[CR7] Alexander M (2010). The new Jim Crow: Mass incarceration in the age of colorblindness.

[CR8] Apel R (2013). Sanctions, perceptions, and crime: Implications for criminal deterrence. Journal of Quantitative Criminology.

[CR9] Ashkanasy NM, Windsor CA, Treviño LK (2006). Bad apples in bad barrels revisited: Cognitive moral development, just world beliefs, rewards, and ethical decision-making. Business Ethics Quarterly.

[CR10] Bachmann LH, Stephens J, Richey CM, Hook EW (1999). Measured versus self-reported compliance with doxycycline therapy for chlamydia-associated syndromes: High therapeutic success rates despite poor compliance. Sexually Transmitted Diseases.

[CR11] Baker T, Gau JM (2018). Female offenders’ perceptions of police procedural justice and their obligation to obey the law. Crime & Delinquency.

[CR12] Bar-Gill O, Harel A (2001). Crime rates and expected sanctions: The economics of deterrence revisited. The Journal of Legal Studies.

[CR13] Barabási A-L (2007). The architecture of complexity. IEEE Control Systems Magazine.

[CR14] Barabási, A.-L. (2016). *Network science*. Cambridge University Press. Retrieved from http://networksciencebook.com.

[CR15] Baron SW (2004). General strain, street youth and crime: A test of Agnew's revised theory. Criminology.

[CR16] Bauhoff S (2011). Systematic self-report bias in health data: Impact on estimating cross-sectional and treatment effects. Health Services and Outcomes Research Methodology.

[CR17] Becker GS (1968). Crime and punishment, an economic approach. Journal of Political Economy.

[CR18] Becker GS (1976). The economic approach to human behavior.

[CR19] Behrend TS, Sharek DJ, Meade AW, Wiebe EN (2011). The viability of crowdsourcing for survey research. Behavior Research Methods.

[CR20] Benson ML, Madensen TD, Pontell HN, Geis G (2007). Situational crime prevention and white-collar crime. International handbook of white-collar and corporate crime.

[CR21] Benson ML, Madensen TD, Eck JE, Simpson S, Weisburd D (2009). White-collar crime from an opportunity perspective. The criminology of white collar crime.

[CR22] Borgatti SP (2005). Centrality and network flow. Social Networks.

[CR23] Borsboom D, Cramer AO (2013). Network analysis: An integrative approach to the structure of psychopathology. Annual Review of Clinical Psychology.

[CR24] Borsboom D, Cramer AO, Schmittmann VD, Epskamp S, Waldorp LJ (2011). The small world of psychopathology. PLoS ONE.

[CR25] Borsboom D, Deserno MK, Rhemtulla M, Epskamp S, Fried EI, McNally RJ, Robinaugh DJ, Perugini M, Dalege J, Costantini G (2021). Network analysis of multivariate data in psychological science. Nature Reviews Methods Primers.

[CR26] Botchkovar EV, Tittle CR, Antonaccio O (2009). General strain theory: Additional evidence using cross-cultural data. Criminology.

[CR27] Braga AA, Weisburd D, Turchan B (2019). Focused deterrence strategies effects on crime: A systematic review. Campbell Systematic Reviews.

[CR28] Bringmann LF, Vissers N, Wichers M, Geschwind N, Kuppens P, Peeters F, Borsboom D, Tuerlinckx F (2013). A network approach to psychopathology: New insights into clinical longitudinal data. PLoS ONE.

[CR29] Chambon, M., Dalege, J., Borsboom, D., Waldorp, L., van der Maas, H., & van Harreveld, F. (2021). Temporal dynamics of compliance and well-being during pandemics: A longitudinal COVID-19 study. PsyArXiv Preprints https://psyarxiv.com/m2spb/.

[CR30] Chambon M, Dalege J, Elberse JE, van Harreveld F (2022). A psychological network approach to attitudes and preventive behaviors during pandemics: A COVID-19 study in the United Kingdom and the Netherlands. Social Psychological and Personality Science.

[CR31] Cialdini RB, Trost MR, Gilbert DT, Fiske ST, Lindzey G (1998). Social influence: Social norms, conformity and compliance. The handbook of social psychology.

[CR32] Cialdini RB, Demaine LJ, Sagarin BJ, Barrett DW, Rhoads K, Winter PL (2006). Managing social norms for persuasive impact. Social Influence.

[CR33] Cilliers P (2000). What can we learn from a theory of complexity?. Emergence.

[CR34] Clarke RV, Bean P (2003). ‘Situational’ crime prevention. Crime: Critical concepts in sociology.

[CR35] Clarke RV, Telley N (2005). Seven misconceptions of situational crime prevention. Handbook of crime prevention and community safety.

[CR36] Coglianese C, Lazer D (2003). Management-based regulation: Prescribing private management to achieve public goals. Law & Society Review.

[CR37] Coglianese C, Nash J, Van Rooij B, Sokol DD (2021). Compliance management systems: Do they make a difference?. The Cambridge handbook of compliance.

[CR38] Cohen LE, Felson M (1979). Social change and crime rate trends: A routine activity approach. American Sociological Review.

[CR40] Dalege J, Borsboom D, van Harreveld F, van der Maas HL (2017). Network analysis on attitudes: A brief tutorial. Social Psychological and Personality Science.

[CR41] Dalege J, Borsboom D, van Harreveld F, van der Maas HL (2018). The attitudinal entropy (AE) framework as a general theory of individual attitudes. Psychological Inquiry.

[CR42] Darley JM, Robinson PH, Carlsmith KM (2001). The ex ante function of the criminal law. Law and Society Review.

[CR43] Decker S, Wright R, Logie R (1993). Perceptual deterrence among active residential burglars: A research note. Criminology.

[CR44] Dieltjens M, Braem MJ, Vroegop AVMT, Wouters K, Verbraecken JA, De Backer WA, Van de Heyning PH, Vanderveken OM (2013). Objectively measured vs self-reported compliance during oral appliance therapy for sleep-disordered breathing. Chest.

[CR45] Donovan JL, Blake DR (1992). Patient non-compliance: Deviance or reasoned decision-making?. Social Science & Medicine.

[CR46] Edelman LB (1992). Legal ambiguity and symbolic structures: Organizational mediation of civil rights law. American Journal of Sociology.

[CR47] Edelman LB, Talesh SA, Parker C, Nielsen VL (2011). To comply or not to comply—that isn’t the question: How organizations construct the meaning of compliance. Explaining compliance: Business responses to regulation.

[CR48] Edelman LB, Petterson S, Chambliss E, Erlanger HS (1991). Legal ambiguity and the politics of compliance: Affirmative action officers' dilemma. Law & Policy.

[CR49] Edwards A (2014). English in the Netherlands: Functions, forms and attitudes.

[CR50] Epskamp S, Borsboom D, Fried EI (2018). Estimating psychological networks and their accuracy: A tutorial paper. Behavior Research Methods.

[CR52] Epskamp S, van Borkulo CD, van der Veen DC, Servaas MN, Isvoranu A-M, Riese H, Cramer AO (2018). Personalized network modeling in psychopathology: The importance of contemporaneous and temporal connections. Clinical Psychological Science.

[CR53] Epskamp S, Waldorp LJ, Mõttus R, Borsboom D (2018). The Gaussian graphical model in cross-sectional and time-series data. Multivariate Behavioral Research.

[CR54] Estévez E, Emler NP (2010). A structural modelling approach to predict adolescent offending behaviour from family, school and community factors. European Journal on Criminal Policy and Research.

[CR55] Feldman Y (2018). The law of good people: Challenging states' ability to regulate human behavior.

[CR56] Feldman Y, Teichman D (2009). Are all legal probabilities created equal?. New York University Law Review.

[CR57] Fine AD, Van Rooij B (2021). Legal socialization: Understanding the obligation to obey the law. Journal of Social Issues.

[CR58] Fine A, Thomas A, van Rooij B, Cauffman E (2020). Age-graded differences and parental influences on adolescents’ obligation to obey the law. Journal of Developmental and Life-Course Criminology.

[CR59] Freeman LC (1978). Centrality in social networks conceptual clarification. Social Networks.

[CR60] Garber MC, Nau DP, Erickson SR, Aikens JE, Lawrence JB (2004). The concordance of self-report with other measures of medication adherence: A summary of the literature. Medical Care.

[CR61] Gau JM (2014). Procedural justice and police legitimacy: A test of measurement and structure. American Journal of Criminal Justice.

[CR62] Gau JM (2015). Procedural justice, police legitimacy, and legal cynicism: A test for mediation effects. Police Practice and Research.

[CR63] Gneezy U, Rustichini A (2000). A fine is a price. Journal of Legal Studies.

[CR64] Gneezy U, Meier S, Rey-Biel P (2011). When and why incentives (don't) work to modify behavior. The Journal of Economic Perspectives.

[CR65] Goldstein NJ, Cialdini RB, Griskevicius V (2008). A room with a viewpoint: Using social norms to motivate environmental conservation in hotels. Journal of Consumer Research.

[CR66] Goodman JK, Cryder CE, Cheema A (2013). Data collection in a flat world: The strengths and weaknesses of Mechanical Turk samples. Journal of Behavioral Decision Making.

[CR67] Google. (2020). *COVID-19 community mobility report: Netherlands, April 17, 2020*. Retrieved from https://www.gstatic.com/covid19/mobility/2020-04-17_NL_Mobility_Report_en.pdf.

[CR68] Gottfredson MR, Hirschi T (1990). A general theory of crime.

[CR69] Grasmick HG, Bryjak GJ (1980). The deterrent effect of perceived severity of punishment. Social Forces.

[CR70] Gul S (2009). An evaluation of rational choice theory in criminology. Girne American University Journal of Sociology and Applied Science.

[CR71] Hansen PG, Larsen EG, Gundersen CD (2021). Reporting on one's behavior: a survey experiment on the nonvalidity of self-reported COVID-19 hygiene-relevant routine behaviors. Behavioural Public Policy.

[CR72] Haslbeck JMB, Waldorp LJ (2020). mgm: Estimating time-varying mixed graphical models in high-dimensional data. Journal of Statistical Software.

[CR73] Henry TR, Robinaugh DJ, Fried EI (2021). On the control of psychological networks. Psychometrika.

[CR74] Hevey D (2018). Network analysis: A brief overview and tutorial. Health Psychology and Behavioral Medicine.

[CR75] Kim PT (1999). Norms, learning and law: Exploring the influences of workers' legal knowledge. University of Illinois Legal Review.

[CR76] Kish-Gephart JJ, Harrison DA, Treviño LK (2010). Bad apples, bad cases, and bad barrels: Meta-analytic evidence about sources of unethical decisions at work. Journal of Applied Psychology.

[CR77] Kooistra EB, Van Rooij B (2020). Pandemic Compliance: A systematic review about influences on social distancing behaviour during the first wave of the COVID-19 outbreak. SSRN Electronic Journal.

[CR78] Lange B (2002). The emotional dimension in legal regulation. Journal of Law and Society.

[CR79] Langton L, Piquero NL (2007). Can general strain theory explain white-collar crime? A preliminary investigation of the relationship between strain and select white-collar offenses. Journal of Criminal Justice.

[CR80] Larsen MV, Nyrup J, Petersen MB (2020). Do survey estimates of the public's compliance with COVID-19 regulation suffer from social desirability bias?. Journal of Behavioral Public Administration.

[CR81] Lunansky G, Naberman J, van Borkulo CD, Chen C, Wang L, Borsboom D (2021). Intervening on psychopathology networks: Evaluating intervention targets through simulations. Methods.

[CR82] McCright AM, Dentzman K, Charters M, Dietz T (2013). The influence of political ideology on trust in science. Environmental Research Letters.

[CR83] McKendall M, DeMarr B, Jones-Rikkers C (2002). Ethical compliance programs and corporate illegality: Testing the assumptions of the corporate sentencing guidelines. Journal of Business Ethics.

[CR84] Meadows DH (2008). Thinking in systems: A primer.

[CR85] Murphy K, Tyler T (2008). Procedural justice and compliance behaviour: The mediating role of emotions. European Journal of Social Psychology.

[CR86] Nagin DS (2013). Deterrence in the twenty-first century. Crime and Justice.

[CR87] Nagin DS, Telep CW (2017). Procedural justice and legal compliance. Annual Review of Law and Social Science.

[CR88] Nolan JM, Wallen KE, Van Rooij B, Sokol DD (2021). Social norms and persuasion. Cambridge handbook of compliance.

[CR89] Nolan JM, Schultz PW, Cialdini RB, Goldstein NJ, Griskevicius V (2008). Normative social influence is underdetected. Personality and Social Psychology Bulletin.

[CR90] O’Fallon MJ, Butterfield KD (2012). The influence of unethical peer behavior on observers’ unethical behavior: A social cognitive perspective. Journal of Business Ethics.

[CR91] Osgood DW, Wilson JK, O’malley PM, Bachman JG, Johnston LD (1996). Routine activities and individual deviant behavior. American Sociological Review.

[CR92] Parker C, Nielsen VL (2009). Corporate compliance systems could they make any difference?. Administration & Society.

[CR93] Paternoster R, Simpson S, Clarke RV, Felson M (1993). A rational choice theory of corporate crime. Routine activity and rational choice: Advances in criminological theory.

[CR94] Peer E, Brandimarte L, Samat S, Acquisti A (2017). Beyond the Turk: Alternative platforms for crowdsourcing behavioral research. Journal of Experimental Social Psychology.

[CR95] Peer E, Rothschild D, Gordon A, Evernden Z, Damer E (2021). Data quality of platforms and panels for online behavioral research. Behavior Research Methods.

[CR96] Pendse SG (2012). Ethical hazards: A motive, means, and opportunity approach to curbing corporate unethical behavior. Journal of Business Ethics.

[CR97] Piquero NL, Sealock MD (2004). Gender and general strain theory: A preliminary test of Broidy and Agnew's gender/GST hypotheses. Justice Quarterly.

[CR98] Polinsky AM, Shavell S, Bouckaert B, De Geest G (2000). Public enforcement of law. Encyclopedia of law and economics, volume V: the economics of crime and litigation.

[CR99] Pratt TC, Cullen FT (2000). The empirical status of Gottfredson and Hirschi's general theory of crime: A meta-analysis. Criminology.

[CR100] Pratt TC, Cullen FT (2005). Assessing macro-level predictors and theories of crime: A meta-analysis. Crime and Justice.

[CR101] Pratt TC, Lloyd K, Van Rooij B, Sokol DD (2021). Self-control and offending. The Cambridge handbook of compliance.

[CR102] Pratt TC, Cullen FT, Sellers CS, Thomas Winfree Jr L, Madensen TD, Daigle LE, Fearn NE, Gau JM (2010). The empirical status of social learning theory: A meta-analysis. Justice Quarterly.

[CR103] R Core Team. (2013). *R: A language and environment for statistical computing*. R Foundation for Statistical Computing. Retrieved from http://www.R-project.org/.

[CR104] Rauscher H, Formanek D, Popp W, Zwick H (1993). Self-reported vs measured compliance with nasal CPAP for obstructive sleep apnea. Chest.

[CR105] Reinders Folmer C, Brownlee M, Fine A, Kuiper ME, Olthuis E, Kooistra EB, de Bruijn AL, van Rooij B (2021). Social distancing in America: Understanding long-term adherence to Covid-19 mitigation recommendations. PLoS ONE.

[CR106] Reisig MD, Bratton J, Gertz MG (2007). The construct validity and refinement of process-based policing measures. Criminal Justice and Behavior.

[CR107] Ridgers ND, Timperio A, Crawford D, Salmon J (2012). Validity of a brief self-report instrument for assessing compliance with physical activity guidelines amongst adolescents. Journal of Science and Medicine in Sport.

[CR108] Robinaugh DJ, Millner AJ, McNally RJ (2016). Identifying highly influential nodes in the complicated grief network. Journal of Abnormal Psychology.

[CR109] Schell-Busey N, Simpson SS, Rorie M, Alper M (2016). What works? A systematic review of corporate crime deterrence. Criminology & Public Policy.

[CR110] Schultz PW, Nolan JM, Cialdini RB, Goldstein NJ, Griskevicius V (2007). The constructive, destructive, and reconstructive power of social norms. Psychological Science.

[CR111] Shavell S (1991). Specific versus general enforcement of law. Journal of Political Economy.

[CR112] Shover N, Hochstetler A (2005). Choosing white-collar crime.

[CR113] Simpson SS, Rorie M, Alper M, Schell-Busey N, Laufer WS, Smith NC (2014). Corporate crime deterrence: A systematic review. Campbell Systematic Reviews.

[CR114] Spano R, Freilich JD (2009). An assessment of the empirical validity and conceptualization of individual level multivariate studies of lifestyle/routine activities theory published from 1995 to 2005. Journal of Criminal Justice.

[CR115] Taylor S, Landry CA, Paluszek MM, Rachor GS, Asmundson GJ (2020). Worry, avoidance, and coping during the COVID-19 pandemic: A comprehensive network analysis. Journal of Anxiety Disorders.

[CR116] Thau S, Derfler-Rozin R, Pitesa M, Mitchell MS, Pillutla MM (2015). Unethical for the sake of the group: Risk of social exclusion and pro-group unethical behavior. Journal of Applied Psychology.

[CR117] Trevino LK, Weaver GR (2001). Organizational justice and ethics program “follow-through”: Influences on employees’ harmful and helpful behavior. Business Ethics Quarterly.

[CR118] Trevino LK, Youngblood SA (1990). Bad apples in bad barrels: A causal analysis of ethical decision-making behavior. Journal of Applied Psychology.

[CR119] Tyler TR (1997). Procedural fairness and compliance with the law. Swiss Journal of Economics and Statistics.

[CR120] Tyler TR (2006). Why people obey the law.

[CR121] Tyler TR (2017). Procedural justice and policing: A rush to judgment?. Annual Review of Law and Social Science.

[CR122] Tyler TR, Blader SL (2005). Can businesses effectively regulate employee conduct? The antecedents of rule following in work settings. Academy of Management Journal.

[CR123] Van Borkulo CD, Van Bork R, Boschloo L, Kossakowski JJ, Tio P, Schoevers RA, Borsboom D, Waldorp LJ (2022). Comparing network structures on three aspects: A permutation test. Psychological Methods.

[CR124] Van de Mortel TF (2008). Faking it: Social desirability response bias in self-report research. The Australian Journal of Advanced Nursing.

[CR125] van der Maas HL, Dalege J, Waldorp L (2020). The polarization within and across individuals: The hierarchical Ising opinion model. Journal of Complex Networks.

[CR126] Van Rooij B, van Rooij B, Sokol DD (2021). Do people know the law? Empirical evidence about legal knowledge and its implications for compliance. Cambridge handbook of compliance.

[CR127] Van Rooij B, Fine A, Van Rooij B, Sokol DD (2021). The opportunity approach to compliance. Cambridge handbook of compliance.

[CR128] van Rooij B, Sokol DD, van Rooij B, Sokol DD (2021). Introduction: Compliance as the interaction between rules and behavior. Cambridge handbook of compliance.

[CR129] Vazsonyi AT, Mikuška J, Kelley EL (2017). It’s time: A meta-analysis on the self-control-deviance link. Journal of Criminal Justice.

[CR130] Walters GD, Bolger PC (2019). Procedural justice perceptions, legitimacy beliefs, and compliance with the law: A meta-analysis. Journal of Experimental Criminology.

[CR131] Weaver GR, Trevino LK, Cochran PL (1999). Corporate ethics programs as control systems: Influences of executive commitment and environmental factors. Academy of Management Journal.

[CR132] Weinberger DA, Schwartz GE (1990). Distress and restraint as superordinate dimensions of self-reported adjustment: A typological perspective. Journal of Personality.

[CR133] Wolfe SE, Nix J, Kaminski R, Rojek J (2016). Is the effect of procedural justice on police legitimacy invariant? Testing the generality of procedural justice and competing antecedents of legitimacy. Journal of Quantitative Criminology.

