# Supplementary material for: A Network Approach to Compliance: A Complexity Science Understanding of How Rules Shape Behavior
Source: J Bus Ethics. 2022 May 10;184(2):479–504. doi: 10.1007/s10551-022-05128-8 (PMC9089293; doi:10.1007/s10551-022-05128-8)
Supplement: Supplementary file 1 — Supplementary file1 (DOCX 573 kb) [file 10551_2022_5128_MOESM1_ESM.docx]

Appendix C Supplementary Material Network Analysis

# Cluster analysis

Similar to (Chambon et al., 2022) clusters in the network were determined through a cluster stability and cluster detection analysis with a cluster walktrap algorithm (see figures below, ^ indicates binary nodes). The stability analysis consisted of 1000 iterations of the cluster analysis, after which we calculated how often different nodes belonged to the same cluster. This resulted in a score between 0 and 1 for each combination of nodes (meaning nodes belonged to the same cluster in no [0] to all [1] iterations). The subsequent cluster detection analysis identified clusters with nodes that belonged to that cluster in over 90 percent of the iterations. Clusters of nodes resulting from the cluster detection analysis resulted in the final clusters.

**Cluster stability**


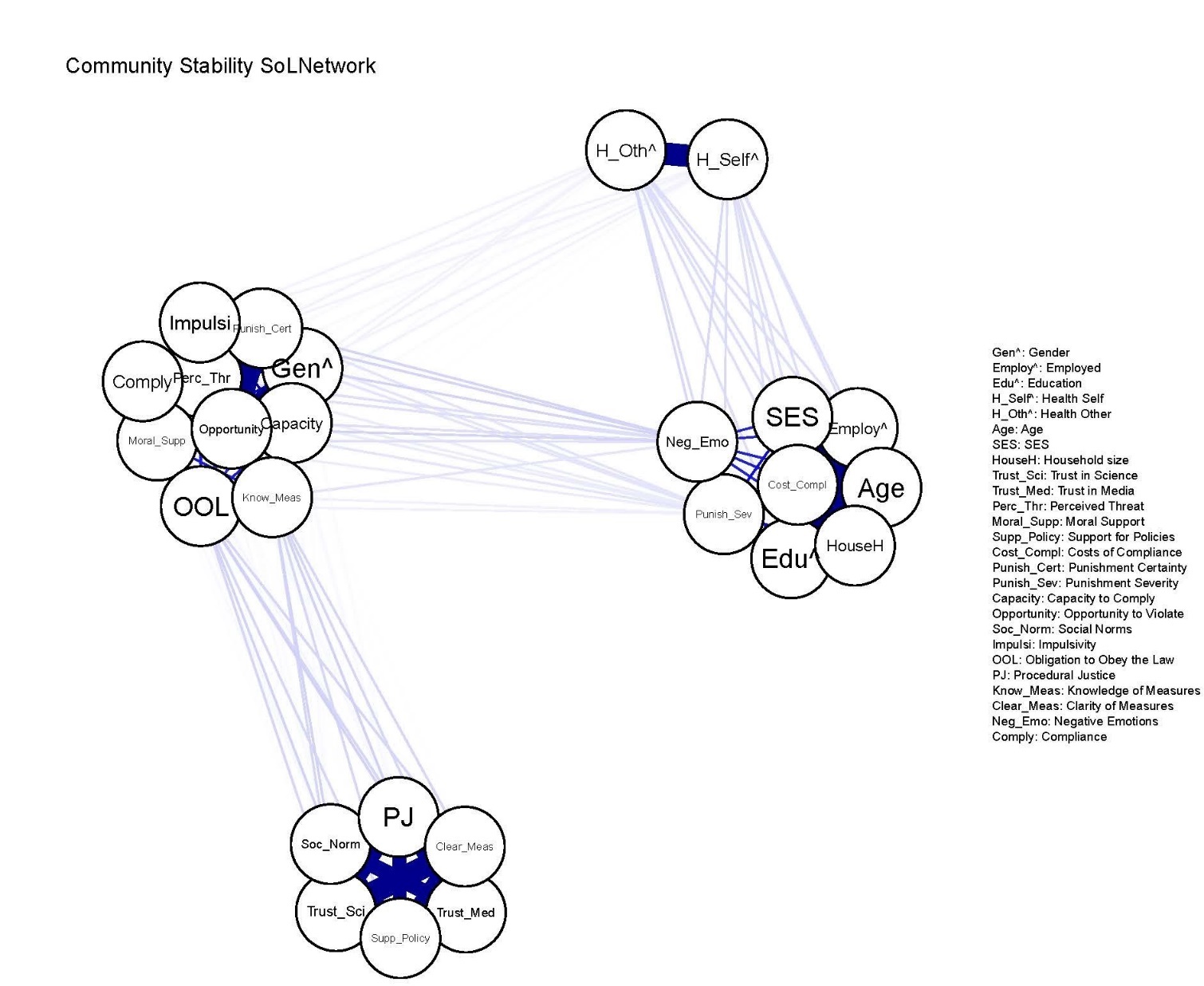


**Cluster detection**

**
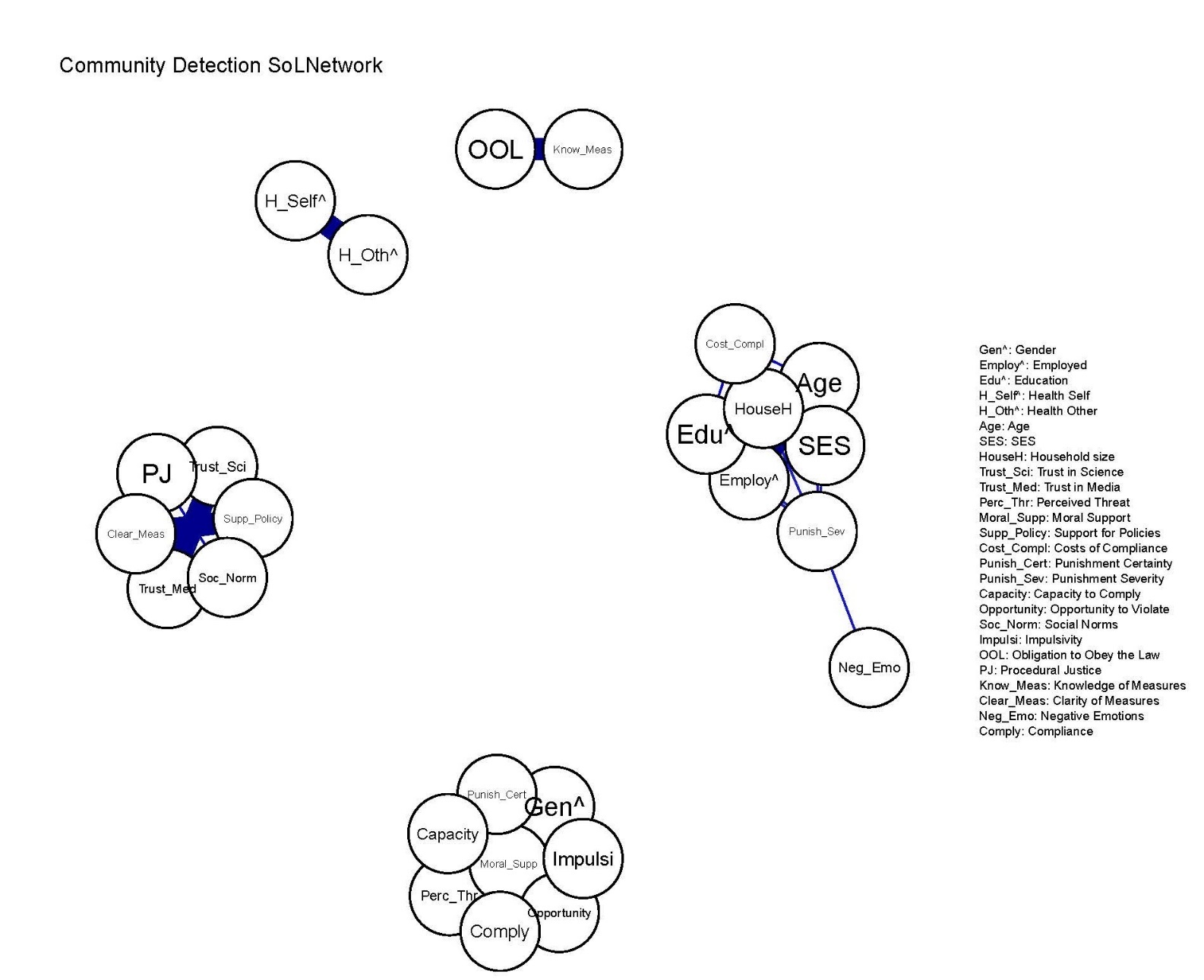
**

# Edge accuracy and difference

The documents are provided [here](https://osf.io/3x5cq/?view_only=fc09f06c7e90459c91515088a05bfe1a).

Edge accuracy: Please note that negative edge weights can appear as positive edge weights in the edge accuracy output, because edge weights are based on networks estimated with mgm (stores edge weights signs separately), whereas edge accuracy analysis is conducted with bootnet (does not include signs from mgm).

Edge difference: *a* = .05, black box indicates significant differences between edges.

# Centrality stability and difference

The documents are provided [here](https://osf.io/3x5cq/?view_only=fc09f06c7e90459c91515088a05bfe1a).

Centrality difference: *a* = .05, black box indicates significant differences between nodes (diagonal box contains node strength).

# R code

The R code is provided [here](https://osf.io/3x5cq/?view_only=fc09f06c7e90459c91515088a05bfe1a). We used the packages *qgraph* (Epskamp et al., 2012) to visualize the graphs, *igraph* (Csardi & Nepusz, 2006) for the cluster detection and *bootnet* (Epskamp, Borsboom, et al., 2018) for the stability and accuracy analyses.
